# Cardiac Ambulatory Monitoring: New Wireless Device Validated Against Conventional Holter Monitoring in a Case Series

**DOI:** 10.3389/fcvm.2020.587945

**Published:** 2020-11-30

**Authors:** Srinivasan Murali, Nicolas Brugger, Francisco Rincon, Manoj Mashru, Stéphane Cook, Jean-Jacques Goy

**Affiliations:** ^1^École polytechnique fédérale de Lausanne Innovation Park, Lausanne, Switzerland; ^2^University Hospital, Fribourg, Switzerland; ^3^Sir Harkisandas Narottamdas Reliance Hospital, Mumbai, India; ^4^Clinique Cecil, Lausanne, Switzerland

**Keywords:** atrial fibrillation, cardiac arrhythmias, electrocardiogram, Holter monitoring, SmartCardia patch, wireless recorder

## Abstract

**Background:** Cardiac arrhythmias are very common but underdiagnosed due to their transient and asymptomatic nature. An optimization of arrhythmia detection would permit to better treat patients and could substantially reduce morbidity and mortality. The SmartCardia ScaAI wireless patch is a novel CE IIa approved, single-lead electrocardiographic (ECG) ambulatory monitor designed for cardiac arrhythmias detection.

**Hypothesis:** The accuracy of the new SmartCardia wireless patch to detect arrhythmias is comparable to the conventional Holter monitoring.

**Methods:** Patients referred for a suspicion of arrhythmia between February and March 2020 were included in the trial. Simultaneous ambulatory ECG were recorded using a conventional 24-h Holter and the SmartCardia. The primary endpoint was the detection of cardiac arrhythmias over the total wear time of the devices, defined as premature atrial contraction (PAC), supraventricular tachycardia ≥3 beats, premature ventricular contraction (PVC), and ventricular tachycardia ≥3 beats. Conduction abnormalities, pause ≥2 s and atrioventricular block (AVB), were also tracked. McNemar's test was used to compare the matched pairs of data from both devices.

**Results:** A total of 40 patients were included in the trial. Over the total wear time, there was no significant difference between the devices for ventricular and supraventricular arrhythmias detection. Pauses and AVB were equally identified by the two devices in three patients.

**Conclusion:** Over the total wear time, the SmartCardia device showed an accuracy to detect arrhythmia similar to the 24-h Holter monitoring: single-lead, adhesive-patch monitoring might become an interesting alternative to the conventional Holter monitoring.

## Introduction

**Figure 1:**
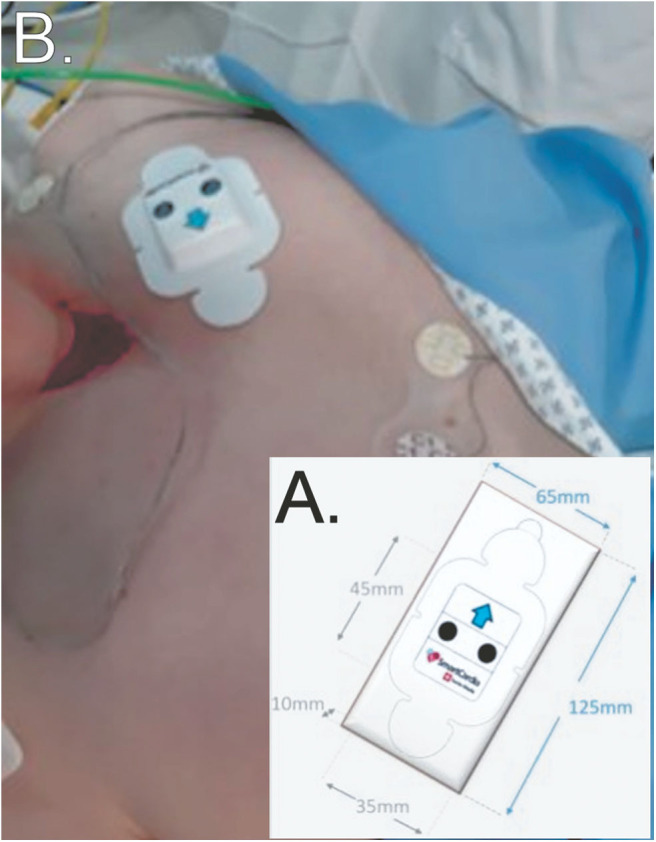
**(A)** Smartcardia Wireless Disposable, **(B)** PatchDevice patched on the patient's chest.

Cardiac arrhythmias, such as atrial fibrillation (AF), are often asymptomatic and sometimes associated with adverse events, such as strokes and peripheral arterial embolisms (1, 2). Twenty-four hour Holter monitoring, introduced in the late 1940s, remains the most widely used method to detect cardiac arrhythmias in ambulatory patients (3) despite a low diagnostic yield, 15–40%, in this patients population (4–6). Moreover, the 24-h Holter monitoring fails to detect the culprit arrhythmia in a significant proportion of symptomatic patients (7). In addition, morbidity and mortality associated with subclinical AF are certainly underestimated and the conventional 24-h monitoring has a low sensitivity for the detection of such arrhythmia (8). Event recorder monitoring can significantly increase this yield, but ~25% of the patients are unable to activate their device during a symptomatic episode (9). It has been recently shown that the ZIO patch, a wireless electrocardiography, allows detection of arrhythmias in children confirming the usefulness of such devices in the daily practice (10). The SmartCardia ScaAI patch (SmartCardia, EPFL Innovation Park, Lausanne, Switzerland) is a single lead lightweight electrocardiographic (ECG) device and wearable patch (Figure 1A) with water-resistant properties. The device is completely wireless, and does not have any external lead wires allowing the patients to participate in their daily routine activities with minimum disturbance. The SmartCardia system is versatile, can be used as a 24-h or long term (7-day) cardiac monitoring in the ambulatory setting, and give alert to physicians via a cloud in case of relevant arrhythmia. The device is placed on the upper left quadrant of the patient's chest (Figure 1B), and can wirelessly communicate the data to a cloud, as well as store them locally on the device. Once monitoring is completed, the data are stored on a cloud and available for the physicians.

We aimed to prospectively evaluate the diagnostic accuracy of this novel adhesive patch monitor compared to the standard 24-h Holter monitoring in patients referred for evaluation of cardiac arrhythmias by ambulatory ECG monitoring: all additional features of the SmartCardia would be “gadgets” as long as the diagnostic accuracy of the device is not proved non-inferior to the gold standard.

## Methods

### Patient Inclusion and Definition of Arrhythmia

The clinical trial was conducted at the HN Reliance Hospital (Mumbai, Maharastra), HFR (Hôpital Cantonal, Fribourg, Switzerland), and Clinique Cecil (Lausanne, Switzerland). The institution's board and ethical committee reviewed and approved the protocol. Between February and March 2020, symptomatic patients (palpitations, dizziness, presyncope, or syncope) referred for ambulatory ECG monitoring were enrolled prospectively in a consecutive fashion after having signed the informed consent. Then, the patients were fitted out with the adhesive patch and a 24-h Holter monitor. Inclusion criteria included an age of ≥18 year-old, being under evaluation for cardiac arrhythmia and able to comply with continuous monitoring. Exclusion criteria were any skin allergies to adhesive or thorax skin irritation. The 24-h Holter monitor and SmartCardia device were activated at the same time for simultaneous cardiac monitoring. A minimum of 24 h of recording was required to be included in the trial and data were anonymously collected and analysis was performed in a double blind manner. Significant arrhythmias or conduction abnormalities were defined as one of the following: premature atrial contraction (PAC), supraventricular tachycardia ≥3 beats, premature ventricular contraction (PVC) and ventricular tachycardia ≥3 beats, pause ≥2 s, and atrioventricular block (AVB).

### Device Description and Evaluation of Arrhythmia

The system is CE-approved: it is a wireless patch with a low-cost disposable component and a re-chargeable/re-usable electronic unit, 65 over 125 mm (Figure 1A). The patch records a single-lead ECG and evaluates heart rate (HR), HR variability, and arrhythmias. The data are transmitted by Bluetooth to a mobile phone or router. The recorded signals and parameters are also stored on the device which is placed on the upper left quadrant of the patient's chest (Figure 1B). The patch-based device offers up to 7-days monitoring and storage and 3.5-days real-time connectivity with the cloud through a smartphone on a single battery charge. The ability to receive, store, and interpret a broad range of signals offers the opportunity to go far beyond monitoring individual parameters. If the patient's vitals reach a pathological value, the system gives an alert on the cloud and the physician can see the real-time parameters and ECG signals. The device is also equipped with a mark event button and patients are instructed to push it in case of symptoms.

During the trial, after application of the patch on the chest by the study coordinator/nurse, the device was activated by the SmartCardia mobile application through Bluetooth connectivity. Once, the device was activated, the real-time ECG of the patient could be seen on the screen and the recording of the data was started. The device was removed after at least 24 h of recording, the wireless disposable patch and electronic unit were separated, the patch was disposed and the data were collected via USB cable to a computer and then transferred to a cardiac technician for analysis, quality assurance, and report generation. The report were uploaded on a secure website and made accessible to the physicians. In this study, the ECG based arrhythmia events captured by the patch were compared with a Holter (CardioMem 3-channel, 7 Leads, GE Healthcare, Chicago, IL, USA) as reference method. The Holter data and ScaAI patch data were independently analyzed in a blinded manner, by two different US board certified cardiac technicians using the Holter software and ScaAI patch software, respectively. The reported arrhythmia events from both systems were further validated by an electrophysiologist. In case of discrepancies between the two devices reports for the same patient, the tracings were manually reviewed by the investigators. Noise was defined as parts of the ECG signal that was not analyzable by the technicians, such as movement artifact or muscle activity in the ECG signal.

The presence of tracings showing any arrhythmia or conduction abnormalities were sufficient to assume that the patient had an arrhythmia or conduction abnormality.

### Safety

The materials and the patches that have skin contact meet the ISO 10993, i.e., the criteria for skin irritation, skin sensitization, and *in vitro* cytotoxicity.

### Sample Size Calculation and Statistics

The primary aim of the study was to evaluate the accuracy of the adhesive patch for the detection of arrhythmias and conduction abnormalities or pauses as compared to the 24-h Holter monitor. The average diagnostic yield for the classical 24-h ECG monitoring for significant cardiac arrhythmias is between 28 and 39% (11, 12). In order to obtain at least 10 patients with significant arrhythmia, the required number of patients in the trial was calculated to be 30. Continuous values were expressed as mean ± standard deviation. The two-tailed McNemar's test was used to compare the significant arrhythmias that were detected by the adhesive patch monitor vs. the 24-h Holter monitoring. Two tailed Bland-Altman plots were used to examine the agreement of HR parameters between the two devices.

## Results

Of the 65 patients screened, 25 declined enrollment. Finally a total of 40 patients (34 male and 6 female) with a mean age of 59 ± 20 years (range 19–81 years) were included in the trial. Complete recording was achieved in all patients without any side-effects from the SmartCardia device, like skin rash. There was no device disconnection or interruption of the recording due to poor skin contact with both systems. Total monitoring time was 1,008 h, 45 min with mean wear time of 26.2 ± 8 h for the adhesive patch and 25.8 ± 8 h for the 24-h Holter monitor (p = NS). In four patients the wear time was prolonged up to 48 h for clinical reasons. Patients were asked whether they would prefer the adhesive patch or the Holter monitoring; 90% (36/40) chose the adhesive patch.

### Performance of the Device

**Figure 2:**
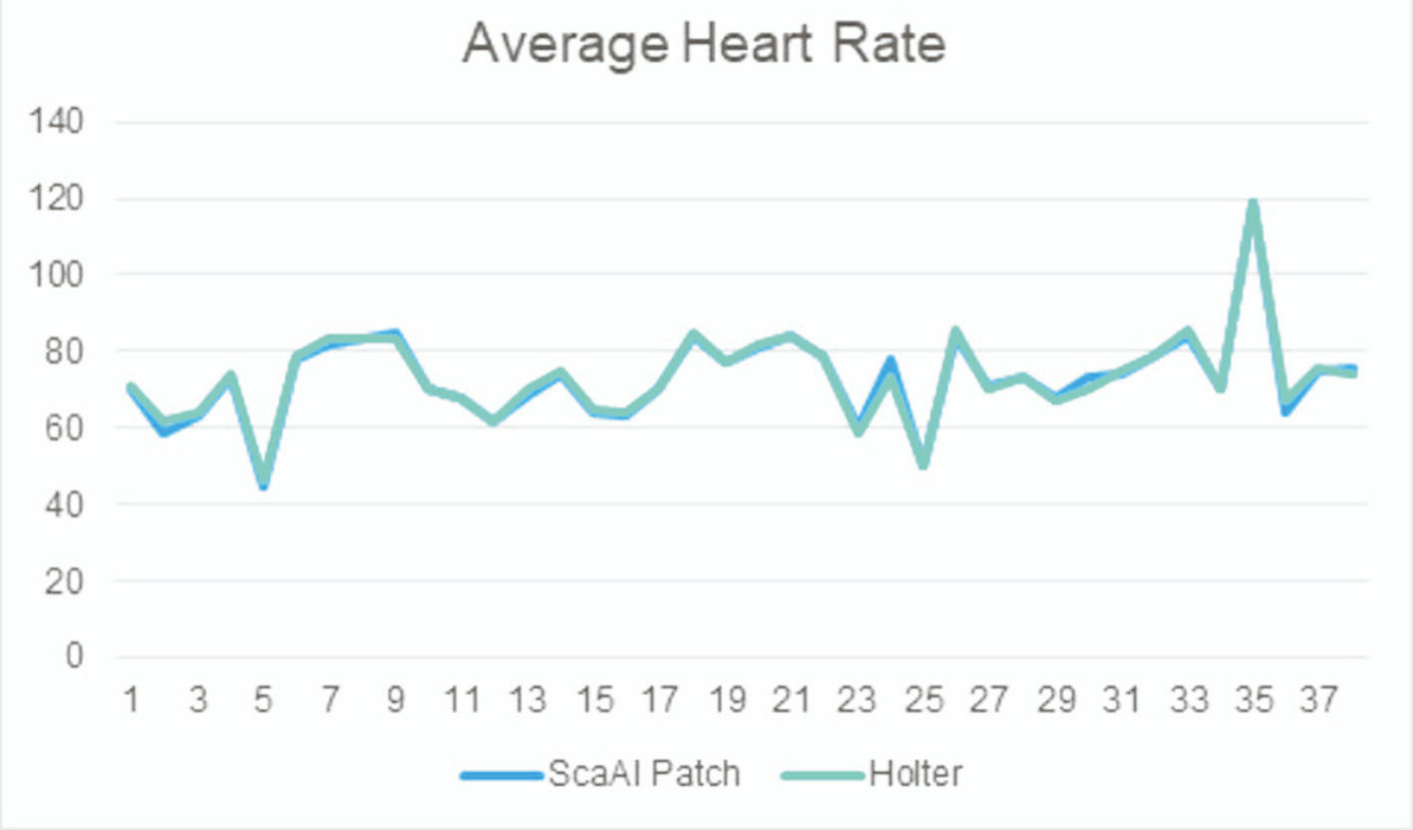
Comparison of the average heart rate between the two systems showing good correlation.

**Figure 3:**
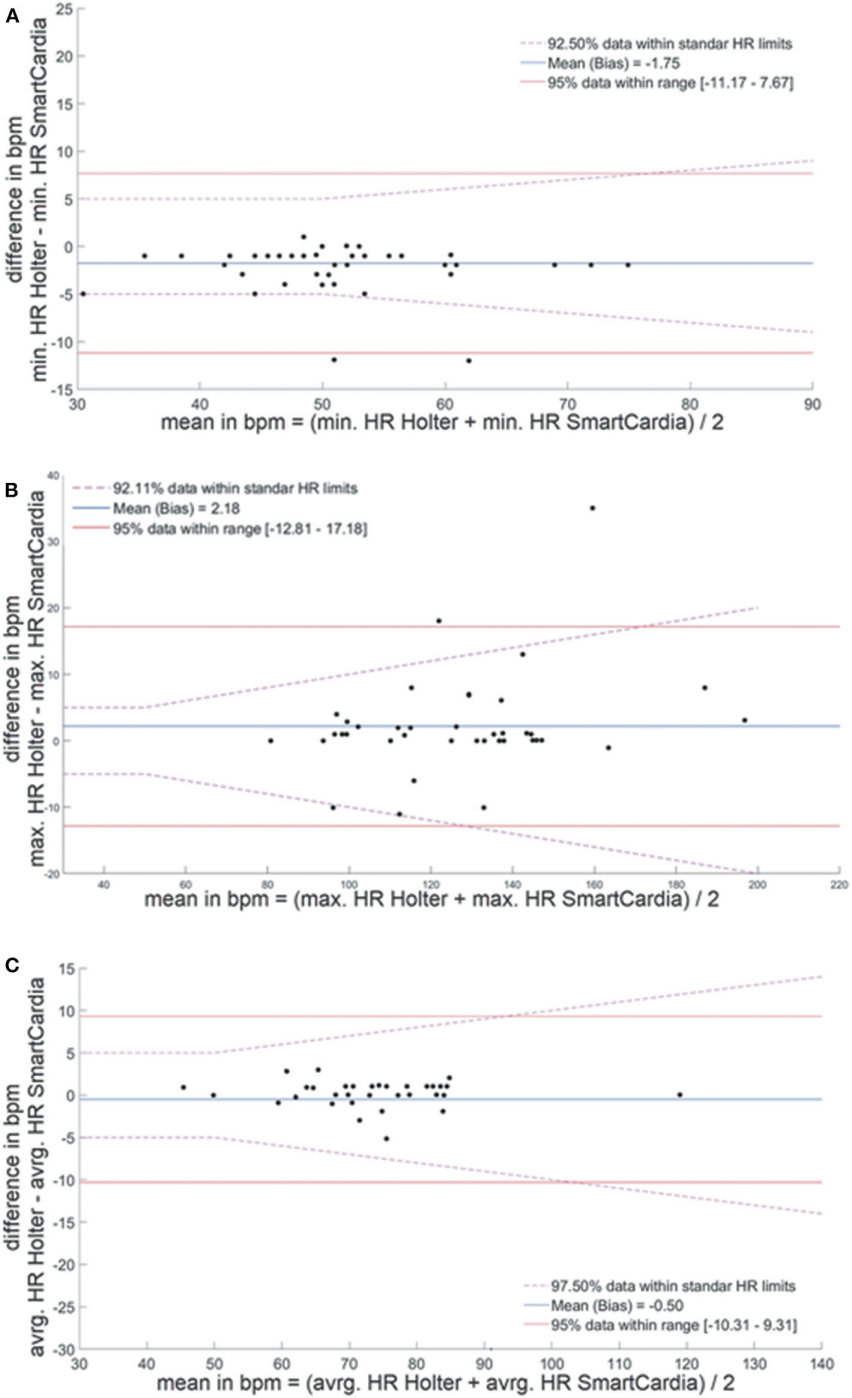
Bland-Altman plots for agreement between heart rate evaluation by the SmartCardia and 24-h Holter monitor. **(A)** Minimal, **(B)** maximal, and **(C)** average heart rate. HR, Heart Rate; BPM, Beat Per Minute; min, minimal; avrg, average; max, maximal.

When device data were compared over the total wear time, we found no significant difference between the two devices in term of average HR, minimal HR, and maximal HR (p = NS). The average noise level time in the entire recordings was slightly higher with the adhesive patch 1.93% (1–8.5) than with the Holter monitoring 1.59% (1–13) but not statistically different (p = NS). The difference for average HR between the Holter monitor and the SmartCardia device was 1 bpm (74 ± 11 bpm vs. 73 ± 10 bpm, p = NS) (Table 1 and Figure 2). For minimal HR the difference was 2 bpm (51 ± 9 bpm vs. 53 ± 8 bpm, p = NS) and for maximal HR the difference was also 1 bpm (125 ± 22 bpm vs. 126 ± 24 bpm, p = NS). The Bland-Altman plots for agreement between the two systems are shown in Figure 3.

**Table 1:** Comparison of the results (heart rate and arrhythmias detection) between the two systems.

	**Holter monitoring**	**SmartCardia**	***p*-value**
Number of patients	40	40	
Noise (%)	1.59	1.93	NS
Average HR (bpm)	74 ± 11	73 ± 10	NS
Minimal HR (bpm)	51 ± 9	53 ± 8	NS
Maximal HR (bpm)	125 ± 22	126 ± 24	NS
PVC	2,858	3,086	NS
Ventricular bigeminy	7	6	NS
Ventricular trigeminy	0	6	<0.01
NSVT	9	9	NS
PAC	410	439	NS
PAC bigeminy	5	4	NS
PAC trigeminy	0	4	<0.01
SVT	6	6	NS
AF	2	2	NS
Pause >2 s	2	2	NS
AVB	1	1	NS

*HR, Heart Rate; PVC, Premature Ventricular Contraction; NSVT, Non Sustained Ventricular Tachycardia; PAC, Premature Atrial Contraction; SVT, Supraventricular Tachycardia; AF, Atrial Fibrillation; AVB, Atrioventricular Block*.

**Figure 4:**
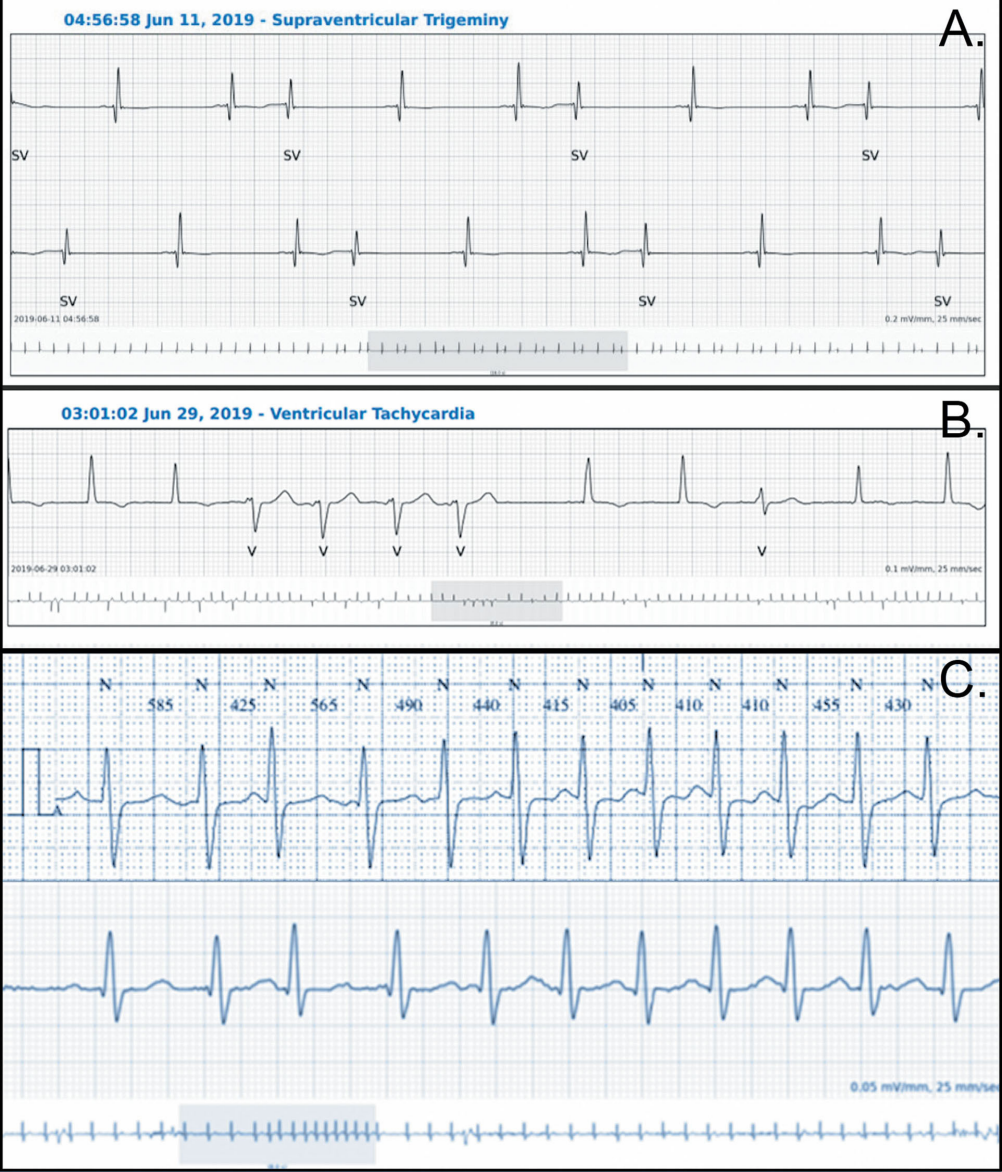
Examples of traces from both devices. **(A)** PAC with some aberrant conduction correctly classified by the SmartCardia (the Holter monitoring classified the premature beats as PVC), **(B)** Short VT correctly classified by the SmartCardia, **(C)** Recording of the same episode of supraventricular tachycardia (above Holter, below SmartCardia).

The average mismatch between the two devices in detecting PVC was 0.25%. The mean incidence of PVC was 2,858 (range 0–50,500) with the adhesive patch and 3,086 (range 0–51,591) with the Holter monitor (p = NS). This small difference is explained by the fact that, in one patient, the Holter monitor detected PVC while the adhesive patch classified these arrhythmias as PAC. Careful analysis of the traces confirmed the supraventricular origin of the arrhythmia (Figure 4A). Ventricular bigeminy was equally detected by the two devices (*n* = 6 for the adhesive patch and seven for the Holter monitor), although ventricular trigeminy was only detected by the adhesive patch (six for the adhesive patch vs. zero for the Holter monitoring) (Table 1). Couplets and a small run of non-sustained ventricular tachycardia occurred in nine patients and were detected by both systems (Figure 4B). Six patients had no PVC or ventricular arrhythmia as evaluated by both systems.

The average mismatch in detecting isolated PAC was 0.03%. The mean incidence of PAC was 439 with the adhesive patch (range 0–2,579) and 410 with the Holter monitor (range 0–2,300) (p = NS). PAC bigeminy was equally detected by the two devices (*n* = 4 for the adhesive patch and five for the Holter monitor), although PAC trigeminy was only detected by the adhesive patch (four for the adhesive patch and vs. zero for the Holter monitoring, Table 1). Run of supraventricular tachycardia were detected by both systems in six patients (Figure 4C) while in two additional patients, a run of supraventricular arrhythmia was not detected, one by the adhesive patch and one by the Holter monitor. AF occurred in two patients and was correctly detected by both devices.

Two significant pauses occurred in two patients during episodes of AF and were detected by both systems with the same duration. AVB occurred in one patient and was correctly detected by both systems.

## Discussion

The SmartCardia adhesive patch is a noninvasive, continuous ambulatory ECG monitoring and is less bulky to wear than the conventional 24-h Holter monitor. Ninety percent of patients found the adhesive patch more comfortable to wear than the 24-h Holter monitoring, and there was no reported skin reaction. The adhesive patch is more user friendly for both the patient and the doctor and it can be sent by mail. In addition, longer monitoring period up to 7 days and real time arrhythmia analysis coupled with physician alert via a cloud offers additional advantages that only make sense if the SmartCardia compares favorably with the conventional Holter monitoring. We demonstrated in this trial a very good correlation with the conventional 24-h Holter monitor and the ability of the adhesive patch to capture arrhythmia, pauses, and conduction abnormalities with a comparable accuracy during the recording time. Other devices, already commercialized, have similar efficiency in arrhythmia detection (13–15). Wireless monitoring has been confirmed as a valuable tool to detect adverse events in high risk patients (14, 16–19). Improvement in the detection of AF have also been shown (20). However, a recent study showed significant differences between wireless sensors (21). For example, the Masimo Radius-7 underestimates HR since it calculates HR from the plethysmographic waveform obtained from the pulse oximeter probe. The EarlySense system may also underestimate HR during periods of arrhythmia since it derives HR from cardiac ballistic movement associated with ejection of blood with each heart cycle. During rapid ventricular rate, ventricular filling is insufficient and as a result, some beats will be undetectable as peripheral pulse. SmartCardia's patch measures and stores ECG and parameters up to 7 days on a single charge. In comparison, Sensium and VitalConnect ECG patches have a limited storage capacity of 10 h or less. Even if there is no direct comparison between the SmartCardia patch and other devices, it seems to have some real and theoretical advantages when compared to the previously cited devices. All the data are stored in a cloud, permitting 24-h a day access to the patient's ECG and it allows continuous monitoring with a high quality signal demonstrated, in the present trial, by the fact that the SmartCardia device gives similar results as the conventional 24-h Holter monitoring, making the device useful for ECG monitoring and arrhythmia detection.

The cost of the device was calculated in order to stay in the range of the current available devices for 24 h or seven days ECGs with a ±5% difference.

Unlike other existing ECG patches, the software allows Holter style reporting of cardiac arrhythmias and offers a visual dashboard for quick analysis of automated alerts. The SmartCardia's patch also performs real time ECG streaming to its cloud platform, where automated arrhythmia analysis is performed.

Recording only one ECG lead could represent a limitation of the ability to detect arrhythmia. However, recent published data (10, 13) showed that two leads does not add significant information when compared to one lead provided the recording is of high quality. The average noise level of the SmartCardia recordings, using only one channel was low and not different from the Holter using three channels. The quality of the signal depends on the adherence and the surface of contact with the patient's skin for both devices and on the agitation of the seven wires for the Holter: For these reasons, it is not likely that the noise reach a clinically significant difference between both devices when using a bigger cohort. Contrary to the conventional 24 h Holter, when disconnection of the SmartCardia occurs, the information is immediately transmitted via the cloud to the physician in charge of the patient.

This study was designed to validate the accuracy of the SmartCardia system. Thus, the sample size and number of events were small but an almost beat to beat comparison of the ECGs was done which substantially increases the quality and the power of the analysis: our results do provide insight into the ability of this wireless sensor to assist in patient monitoring and show that supraventricular and ventricular arrhythmia and ectopic beats were detected by the SmartCardia device as accurately as by the classical Holter monitor. The same was true for pauses and AVB.

Further studies will be conducted in order to demonstrate the advantages of the SmartCardia like longer monitoring time, continuous monitoring, real time automatic arrhythmia analysis, and transmission of clinical significant arrhythmia to physicians.

## Limitations

The major limitation of this study is the small size of the cohort. However, we are confident in the reliability of the device, since it has been tested in more 2,000 patients without problems of data transmission. Indeed, real-time connectivity depend on a stable broadband internet connection, a parameter which could still limit a large scale use of this device.

## Conclusions

Over the total wear time, the SmartCardia device showed an accuracy to detect arrhythmia similar to the 24-h Holter monitoring. Based on these findings, this novel single lead adhesive patch could be a serious alternative to the conventional Holter monitoring in patients referred for ambulatory ECG monitoring.

## Data Availability Statement

The raw data supporting the conclusions of this article will be made available by the authors, without undue reservation.

## Ethics Statement

The studies involving human participants were reviewed and approved by Commission cantonale d'éthique de la recherche sur l'être humain du canton de Vaud (CER-VD) and IEC of Sir H. N. Reliance Foundation Hospital and RC Sir H. N. Reliance Foundation Hospital and Research Centre, Raja Ram Mohan Roy Road, Prarthana Samaj, Girgaum Mumbai. The patients/participants provided their written informed consent to participate in this study.

## Author Contributions

SM and FR: development of the SmartCardia device, design of the study and redaction of the protocol, and revision of the manuscript. NB: conception and design of the study, revision of the manuscript, and formatting and submission of the manuscript. MM: selection of the patients, data collection, and evaluation of the traces of the devices. SC: selection of the patients, data collection, evaluation of the traces of the devices, and revision of the manuscript. J-JG: design, conception, and supervision of the study, data collection, and redaction of the manuscript. All authors contributed to the article and approved the submitted version.

## Conflict of Interest

SM, FR, and MM are members of the board of SmartCardia. The remaining authors declare that the research was conducted in the absence of any commercial or financial relationships that could be construed as a potential conflict of interest.
